# Magnetic melamine cross-linked polystyrene-alt-malic anhydride copolymer: Synthesis, characterization, paclitaxel delivery, cytotoxic effects on human ovarian and breast cancer cells

**DOI:** 10.32604/or.2024.054487

**Published:** 2025-02-28

**Authors:** RAZIEH MOMEN-MESGIN, JAFAR REZAIE, VAHID NEJATI, PEYMAN NAJAFI MOGHADAM

**Affiliations:** 1 Department of Biology, Urmia University, Urmia, 5756151818, Iran; 2 Solid Tumor Research Center, Cellular and Molecular Medicine Research Institute, Urmia University of Medical Sciences, Urmia, 5714783734, Iran; 3 Department of Chemistry, Urmia University, Urmia, 5756151818, Iran

**Keywords:** Breast cancer, Ovarian cancer, PSMA/Me Fe_3_O_4_ MNPs, Paclitaxel (PTX)

## Abstract

**Objectives:**

Due to systematic side effects, there is a growing interest in nanoparticle formulation of anticancer drugs. Here, we aimed to synthesize poly (styrene-alt-maleic anhydride) cross-linked by melamine (PSMA/Me) and coated with magnetite nanoparticles (MNPs) PSMA/Me/Fe_3_O_4_. In addition, we aimed to load paclitaxel (PTX) into PSMA/Me/Fe_3_O_4_ for drug delivery and anticancer investigations.

**Methods:**

Novel PSMA/Me was synthesized via free radical copolymerization, coated with Fe_3_O_4_, and then used as a transporter for PTX delivery. Fabricated copolymer was characterized using SEM, TGA, and XRD techniques. Drug release rate and loading efficiency were investigated. Human ovarian cancer cells (Skov-3) and breast cancer cells (MCF-7 cells) were incubated with the serial concentration of either free PTX or PSMA/Me/Fe_3_O_4_/PTX for cell viability and IC_50_ analysis for 24 and 48 h.

**Results:**

Characterization methods confirmed PSMA/Me copolymer formation. The results showed a significant encapsulation efficiency of 83%. The drug release analysis exhibited that PSMA/Me/Fe_3_O_4_/PTX may be considered pH-sensitive nanocarriers. PSMA/Me/Fe_3_O_4_/PTX reduced cell viability both dose and time-dependently (*p* < 0.05). IC_50_ values of PSMA/Me/Fe_3_O_4_/PTX were low when compared to free PTX either 24 or 48 h post-treatment.

**Conclusions:**

Our results indicated that PSMA/Me/Fe_3_O_4_/PTX was more cytotoxic than PTX in both cancer cells. Findings indicated the potential of PSMA/Me/Fe_3_O_4_/PTX as an anticancer nanocarrier system.

## Introduction

Today, with the advancement of technology, various methods have been designed for targeted drug delivery. Nanoparticles are a major milestone for cancer nanomedicine and drug delivery systems due to their properties of reducing cardiotoxicity, prolonged residence time in human plasma, and targeted delivery to tumors [1,2]. Polymers have a wide range of applications in biotechnology due to the increased solubility of nanoparticles, and the chemical surface modification of synthetic macromolecular drug carrier systems [3,4]. Poly (styrene-alt-maleic anhydride) (PSMA) is a well-known copolymer that enhances signaling molecule loading. It has shown remarkable performance in drug delivery and enzyme immobilization, especially for anticancer purposes [5]. The performance of copolymers in the drug delivery system is very efficient due to the creation of 3 nm pores in the nanoparticles due to the increase in the surface-to-volume ratio. Therefore, we chose PSMA, as our model to develop a targeted drug delivery system. The function of the PSMA copolymer in the drug delivery system is to encapsulate hydrophobic drugs such as paclitaxel (PTX) within its hydrophobic interior, allowing for direct release of the drug without the need for conversion to a prodrug, reaching the tumor site [6]. Most studies have shown that PSMA can transport drugs inside itself and reduce their cytotoxicity in biological systems. It also increases the circulation time of the drug in the bloodstream and thus results in higher delivery efficiency [6]. Magnetic nanoparticles (MNPs) utilize the heat generated when exposed to an alternating magnetic field (AMF). Its significant advantage is due to penetrating deep tissue and destroying cancer cells.

Melamine has long been used as a three-dimensional agent in the preparation of coatings and composites. Melamine cross-linkers have been shown to improve chemical resistance, hardness and exterior durability [7,8]. Due to the availability and low price of this raw material, it has attracted the attention of research and industrial groups. Because of these features, we also decided to use this material in the preparation of composites used in the pharmaceutical industry [7,8]. PTX from the Taxane family is one of the most commonly used drugs to treat high-risk cancers [9]. Due to the insensitivity of PTX to tumor tissues, its use is limited because it causes damage to normal tissues. In addition, its poor solubility in water has limited its direct use. Due to their special characteristics, drug-carrying nanoparticles reduce the toxicity and accumulation of this drug and increase the durability of the drug [10]. In addition, nanocarriers can be targeted to tumor tissues through the enhanced permeation and retention effect (EPR), ultimately leading to superior clinical efficacy [11,12]. According to global cancer statistics, ovarian cancer is the seventh most prevalent form of cancer among malignant tumors and ranks as the eighth leading cause of cancer-related death in women worldwide [13]. Ovarian cancer has the highest mortality rate among women and is only surpassed by cervical and uterine cancer in terms of mortality rates. It’s worth noting that the incidence of this cancer differs depending on the country and ethnicity [14,15]. Various reproductive and hormonal factors can affect the risk of ovarian cancer. Surgery plays an important role in the treatment of ovarian cancer, as it can aid in diagnosis and staging, even for advanced cases. Initial treatment with chemotherapy drugs has been effective in most patients. In addition, new methods of delivering chemotherapy (such as PTX) through the intraperitoneal route have increased survival rates [16,17]. However, despite initial positive responses to chemotherapy, ovarian cancer patients often experience recurrence. Fortunately, new targeted biological agents such as nanoparticle drug delivery show potential in inhibiting cell growth, giving hope for better cancer therapy [18]. Another common cancer among women is breast cancer, which is the second leading cause of cancer-related deaths in women worldwide [19,20]. Chemotherapy of this disease, which includes taxanes such as docetaxel and PTX, binds to microtubules and prevents their separation, which leads to cell cycle arrest and apoptosis [21]. In this work, we aimed to synthesize the modified PSMA copolymer, which was coated with Fe_3_O_4_ MNPs, and then load PTX within this copolymer to deliver it to cancer cells. In addition, we investigated the cytotoxic effect of PSMA/Me/Fe_3_O_4_/PTX on both breast cancer (MCF-7) and ovarian cancer (Skov-3) cells, respectively.

## Materials and Methods

### Materials

Maleic anhydride (MA, CAS No. 108-31-6), Melamine (Me, CAS No. 108-78-1), and Methyl Thiazolyl diphenyl Tetrazolium bromide (MTT, CAS No. 298-93-1) were provided from Sigma-Aldrich, Merck KGaA, Darmstadt, Germany. Benzoyl peroxide (BPO, CAS No. 94-36-0), Paclitaxel (PTX, CAS No. 33069-62-4), penicillin-streptomycin (Pen-Strep, CAS No. 3810-74-0), and styrene (ST, CAS No. 100-42-5) were provided from Merck (Merck KGaA, Darmstadt, Germany). Before use, ST was purified by distillation at reduced pressure. Solvents and other chemicals such as tetrahydrofuran (THF, CAS No. 401757), dimethylformamide (DMF, CAS No. 68-12-2), methanol (CAS No. 67-56-1), Dimethyl Sulfoxide (DMSO, CAS No. 67-68-5) were purchased from Sigma Aldrich (St. Louis, MO, USA). RPMI (Gibco, Catalog No. 11875093) and Fetal Bovine Serum (FBS, Catalog No. 35050061) were provided from Gibco, Thermo Fisher Scientific, Waltham, MA, USA.

### Instruments

The surface morphology of the synthesized sample and the drug release characteristics were studied on a scanning electron microscope (FE-SEM, Joint Stock Company, Tescan, Brno, Czech Republic) and ultraviolet-visible spectrophotometer (UV-Vis, Biomate5, Thermo Fisher Scientific Inc., Waltham, MA, USA), respectively. X-ray diffraction (XRD) pattern was recorded using XRD machine (PHILIPS, PW1730, Netherland). To obtain thermogravimetric analysis (TGA) profiles using TGA analyzer system (TGA STA6000, PerkinElmer, Inc., Shelton, CT, USA).

### Preparation of PSMA

The PSMA sample was prepared according to previously reported literature with some adjustments [22]. In a 100 mL flask with a magnetic stirrer, 2 g of maleic anhydride and 2.32 g of styrene, measured with a pipette, were mixed in 50 mL of THF solvent. The mixture was stirred under nitrogen gas at 0°C–5°C for 20 min. Then, 0.0197 g of azobisisobutyronitrile (AIBN) was added, and the mixture was kept at 80°C for 7 h. After the reaction, the volume of THF was doubled, methanol (30 mL) was added, and the mixture was filtered (30 µm, Smooth Paper SM-180) to obtain a white precipitate of the desired copolymer. The obtained sediment was left to dry completely at 30°C for 24 h [23].

### Preparation of me-cross-linked PSMA

The method of modification of PSMA with Me is as follows: 1 g of PSMA was mixed with 50 mL of distilled water. The mixture was then stirred at 50°C for 15 min to form a homogeneous emulsion. Then, Me solution (1.13 g of Me in 50 mL of distilled water with 5 mL of 0.1 M hydrochloride) was added to the mixture and after 4 h of reflux at 50°C, 2N hydrochloride solution was added to the mixture until the pH reached 4–5 using a pH meter. As a result, a white product with a gelatin-like consistency was obtained. The final product was filtered and dried under a vacuum drying oven at room temperature [23].

### Synthesis of magnetic nanoparticles (PSAMA/Me/Fe_3_O_4_)

To synthesis PSMA/Me/Fe_3_O_4_ magnetic nanoparticles firstly, Fe_3_O_4_ MNPs were synthesized. For this purpose, in a 250 mL flask, 100 mL of deionized water 5.84 g (0.0216 moL) of Fe (III) and 2.17 g (0.0108 moL) of Fe (II) chloride were added under nitrogen gas and stirred for 3 h at 40°C. Then 10 mL of 25% ammonia was added at 80°C for 30 min under magnetic stirrer [24]. The precipitate was collected by magnet and washed several times with ionized water and dried to constant weight under vacuum. In the second step, 1 g PSMA/Me was added to a 50 mL H_2_O/ethanol (50:50 v %) solvent and stirred to obtain a homogeneous mixture. Then 0.4 g Fe_3_O_4_ nanoparticles was added and stirred overnight. The precipitate was collected by an external magnet and washed with deionized water. The obtained PSMA/Me/Fe_3_O_4_ composites were dried under a vacuum to reach a constant weight.

### PTX loading-release assay

To accurately load the anticancer drug PTX into the PSMA/Me copolymer, a series of crucial steps were meticulously followed. First, 1.2 g of PSMA was precisely added to a solution containing PTX at a concentration of 6000 ppm in 12 mL of solvent. The mixture was stirred for 48 h in a light-protected environment at room temperature, at a speed of 80 rotations per minute, until the drug encapsulation reached equilibrium. Following this, the supernatant of PSMA/Me loaded with PTX (PSMA/Me/PTX) was retained after purification to determine the adsorption capacity of PSMA/Me using UV-Vis spectroscopy. Then, based on the obtained absorption values, the encapsulation efficiency (EE%) and loading capacity (LC%) were calculated using the following equations. Subsequently, the product was washed twice with distilled water to remove the free drug, and the product sediment was used for cell efficiency testing. Encapsulation efficiency (EE%) = [(weight of initial drug added-weight of unreleased drug in the supernatant)/weight of initial drug added] × 100.

Loading efficiency (LE%) = [(weight of initial drug added–weight of “unentrapped drug” free in the supernatant)/weight of PSAM] × 100.

To investigate the release profiles of PTX from the PSMA/Me copolymer, a mixture was prepared in a 50 mL Erlen-Meyer flask containing 25 mL of buffer solution. Two distinct pH levels were utilized: 5, 6.5, and 7.4. The experiment was conducted at room temperature. To determine the percentage of PTX released over time, 2 mL aliquots of the buffer solution were periodically (every 30 min) withdrawn from the flask and returned to the flask after determining the concentration. These aliquots were subsequently replaced with an equal volume of fresh buffer solution to maintain a constant medium volume throughout the experiment. The collected aliquots were subjected to analysis using a UV-Vis spectrophotometer. The absorbance of each sample was measured at λ_max_ = 235 nm to determine the concentration of PTX present in the released fraction. By monitoring the PTX concentration over time, the release kinetics and profile of the drug from the PSMA/Me copolymer could be assessed.

### Cell culture

MCF-7 (NCBICode: C135, Pasteur Institute of Iran, Tehran, Iran) and Skov-3 (NCBICode: C209, Pasteur Institute of Iran) human breast and ovarian cancer cell lines were donated from Tabriz University of Medical Sciences, Tabriz, Iran. They were checked for contaminations regarding mycoplasma contamination and were cultured in RPMI 1640 medium supplemented with 10% fetal bovine serum (FBS) and 1% streptomycin/penicillin. The cells were incubated in a controlled incubator. The incubation condition was 37°C with 95% humidity and 5% CO_2_. Upon 80% confluency, cells were subcultured into proper T25 and T75 tissue culture flasks using trypsin-EDTA (Gibco) [25].

### Cytotoxicity assay

Cells were initially seeded in 96-well tissue culture plates at a density of 7 × 10^3^ per well and incubated for 24 h in RPMI 1640 containing 10% FBS and streptomycin/penicillin 1%. The cytotoxic effects of free drug (PTX) and PSMA/Me/Fe_3_O_4_/PTX were evaluated by exposing the cells to varying concentrations of PTX or PSMA/Me/Fe_3_O_4_/PTX (1, 2, 4, 8 μM) for 24 and 48 h in RPMI 1640 containing 10% FBS and streptomycin/penicillin 1%. A control group that did not receive any treatment was included for each set. Following the treatment, MTT solution (5 mg/mL) was added to each well and kept at 37°C for 4 h. Next, the cell medium was removed, and formazan was dissolved in DMSO (Sigma-Aldrich) at room temperature. The absorbance (optical density) was measured at 570 nm using an ELISA reader instrument (BioTek800 TS), and cell viability was calculated relative to the control cells. The IC_50_ value, representing the concentration of PTX or PSMA/Me/Fe_3_O_4_/PTX required to inhibit cell growth by 50%, was also determined [26].

### Statistical analysis

Data was reported as Means± SD of three sets of experiments. GraphPad Prism (GraphPad Software, Inc., Boston, MA, USA, Ver. 8) was used to analyze data by One-way ANOVA and Tukey *post-hoc* test. *p* < 0.05 was considered statically significant.

## Results

### Preparation and characterization

**Figure 1:**
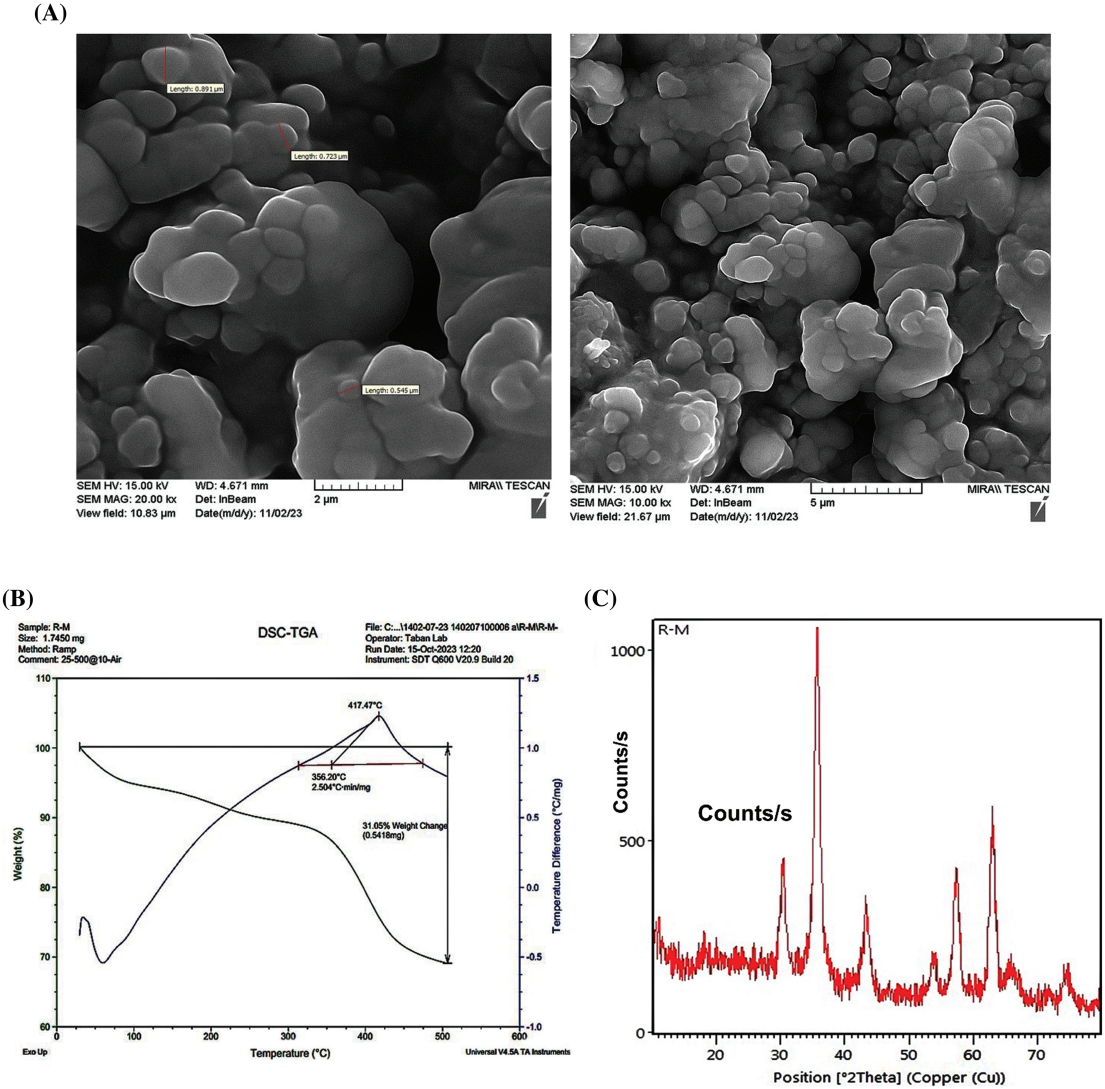
Characterization methods for PSMA/Me/Fe_3_O_4_. (A) Scanning electron microscopy (SEM). (B) Thermal gravimetric analysis (TGA). (C) The X-ray Diffraction (XRD).

The surface morphology of the synthesized (PSMA/Me/Fe_3_O_4_) magnetic nanoparticles was studied by SEM technique and the obtained images are shown in Fig. 1A. As can be seen, the obtained PSMA/Me/Fe_3_O_4_ illustrated an uneven surface with spherical shapes contains holes between granular moieties that can be useful in drug encapsulation. Compared to TEM, SEM costs less to procure, takes less time to generate an image, requires less time for specimen preparation, and accepts thicker samples that are much larger. Thermal gravimetric analysis (TGA) was performed to study the polymeric materials’ thermal stability. As can be seen in Fig. 1B, from ambient temperature to approximately 100°C, there is a gradual decrease in weight, indicative of low-level volatilization or moisture loss (surface water). However, the most notable change occurs at 350°C, with a sharp decrease in weight, suggesting a significant transformation or decomposition process of the copolymer network. This abrupt shift likely corresponds to a thermal degradation or decomposition event, leading to a rapid release of volatile components or breakdown of molecular structures. Despite indications in the research suggesting complete degradation of the copolymer network at 400°C, only approximately 30% of it seems to have been lost, likely attributed to Fe_3_O_4_ nanoparticles. Overall, throughout the analysis, the material undergoes a weight loss of up to 30%, indicating a considerable degree of thermal instability or decomposition within the specified temperature range and weight loss occurs continuously in two steps up to 500°C.

### The X-ray diffraction (XRD)

The XRD pattern obtained for the PSMA-Fe_3_O_4_ composite provides valuable insights into its crystalline structure and phase composition (Fig. 1C). The pattern exhibits distinctive peaks corresponding to the crystalline phases present in the composite such as 2θ = 30, 35, 43, 53, 57, 63 peaks. It seems that the crystalline phase in the composite is influenced by the crystalline nature of magnetite nanoparticles and has face-centered cubic lattice structures. The Fe_3_O_4_ associated with PSMA are discernible, indicating the presence of both constituents in the material, and these peaks are characterized by their positions, intensities, and widths, reflecting the crystallinity and arrangement of atoms within the composite and well-matched with the reference pattern of Fe_3_O_4_ [25].

### Fourier transform infrared (FT-IR) spectroscopy

**Figure 2:**
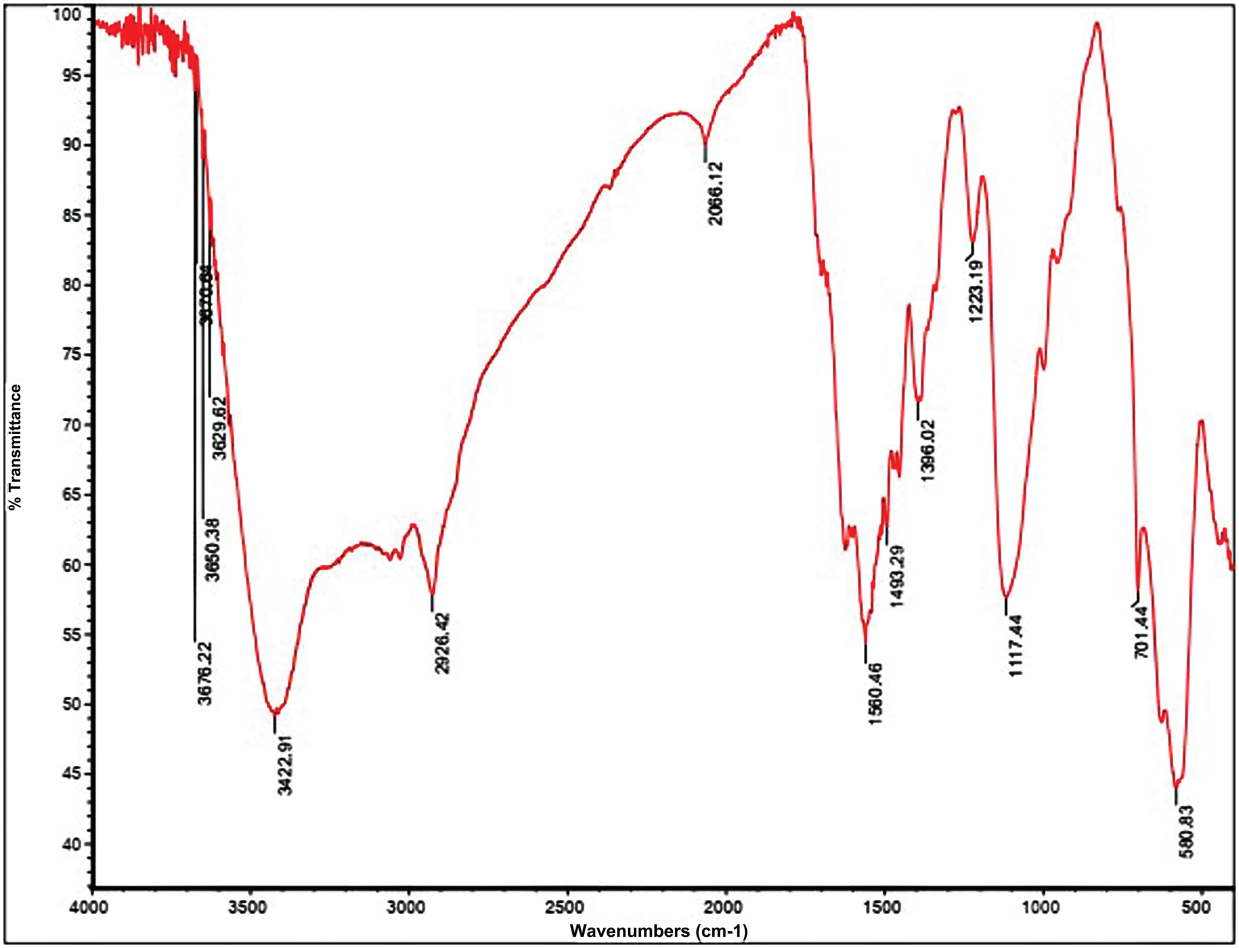
Fourier Transform Infrared Spectroscopy (FTIR) analysis for PSMA/Me/Fe_3_O_4_.

The synthesized samples were studied by FT-IR analysis and the FT-IR spectrum of final PSMA/Me/Fe_3_O_4_ was depicted in Fig. 2. The interpretation of all FT-IR spectra is as follows:

The FTIR spectrum of PSMA reveals distinct absorption bands indicative of its molecular composition and structural features. Within the spectrum, absorption bands are observed between 1450–1500 cm^−1^ and 580–760 cm^−1^, corresponding to the stretching of C=C bonds and the bending of C-H bonds in the aromatic ring of the styrene repeated groups. Additionally, strong absorption bands are evident around 1779 ± 1 cm^−1^ and 1855 ± 1.2 cm^−1^, attributed to the carbonyl stretching of the anhydride groups (C=O) in the maleic anhydride (MA) repeated groups. Notably, characteristic absorption bands at 1200–1300 cm^−1^, associated with cyclic C-O stretching in the MA residue, are also detected in the PSMA sample. This suggests the presence of unreacted anhydride rings following polymerization. The simultaneous presence of aromatic alkene (1494, 1603 cm^−1^) and anhydride carbonyl (1779, 1855 cm^−1^) bands in the IR spectrum of PSMA confirms the successful copolymerization between Styrene and MA comonomers. Furthermore, the absence of the C=O anhydride ring (1779, 1855 cm^−1^) and the broadening of peaks around 1620 ± 1.1 cm^−1^ indicate the conversion of cyclic anhydride groups to amidic carbonyl groups following PSMA amidification with melamine molecules. According to the PSMA-Fe_3_O_4_ spectrum, the width of the peaks in the region of 3000 to 3500 cm^−1^ has broadened due to the O-H group, and the peaks have appeared in the region of 580 cm^−1^ are related to the Fe-O group bound in Fe_3_O_4_. It confirms the establishment of Fe_3_O_4_ nanoparticles on PMSA. The interpretation of PSMA and the PSMA grafted by melamine spectra is by our published article [23].

### PTX loading and release assays

**Figure 3:**
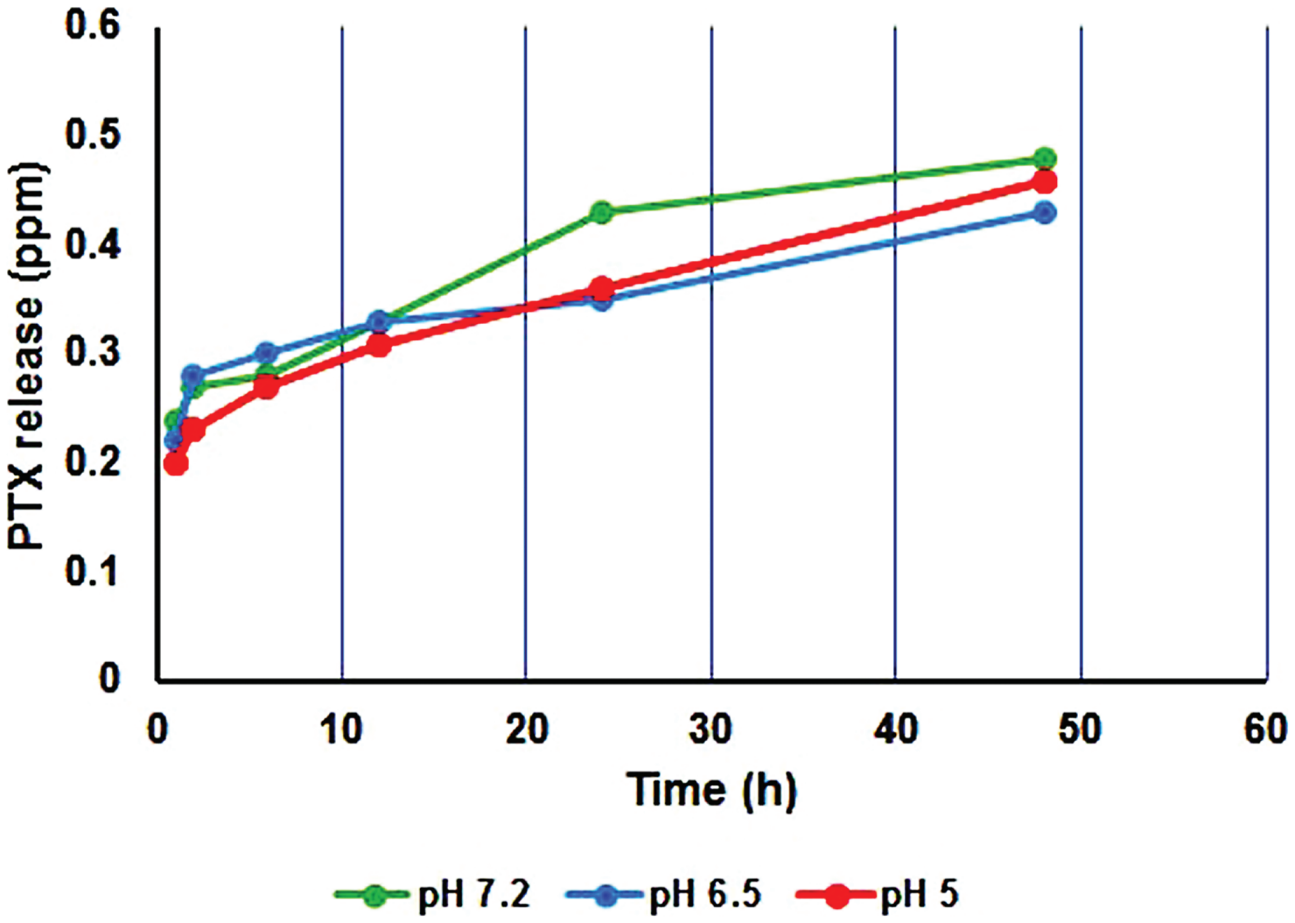
Release profile of PTX from PSMA/Me/Fe_3_O_4_/PTX during 48 h.

The presence of Me as a cross-linker in the copolymer structure not only may improve the mechanical property but also enhance the drug encapsulation efficiency (EE% = 83%) because of making porosity in the copolymer network. In addition, the LE for PSMA/Me/Fe_3_O_4_ was 78% according to the formula. The PSAM/Me/Fe_3_O_4_ drug release profile was studied in acidic (pH = 5 and 6.5) and neutral (pH = 7.2) media using PTX as a therapeutic drug model. The PTX release from PSAM/Me/Fe_3_O_4_ is shown in Fig. 3. The PTX release was 0.46, 0.43, and 0.48 ppm at pH 5, 6.5, and 7.2, respectively after 48 h. As a result, the PTX release from this polymer in neutral media was higher than in other media. Of course, there are differences between drug release in an acidic environment and a basic one. The reason for this difference can be due to the protonation of functional groups in the polymeric network and drug structure it weakened the interaction between the polymeric network and drug molecules and the fast release was seen in an acidic medium.

### Cytotoxic effects of PSMA/Me/Fe_3_O_4_/PTX

**Figure 4:**
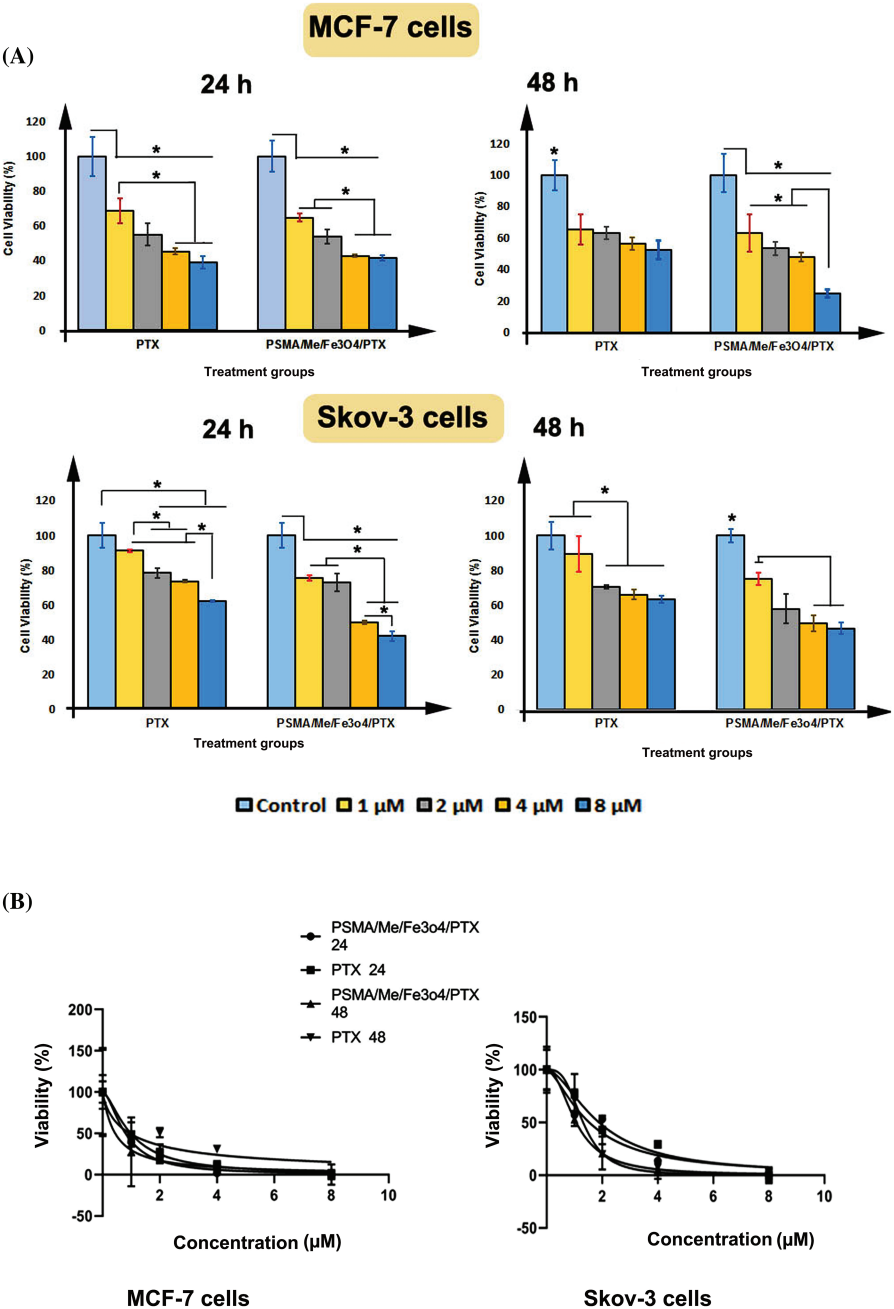
Percentage of cell viability was measured through MTT assay. (A) Both MCF-7 cells and Skov-3 cells were treated with either PTX or PSMA/Me/Fe_3_O_4_/PTX for 24 and 48 h. (B) The curves of IC50 concentration of PTX and PSMA/Me/Fe_3_O_4_/PTX for 24 and 48 h. Data were presented as Means ± S.D of three sets of experiments at least. Data were analyzed with One-way ANOVA and Tukey *post-hoc* test. *p < 0.05.

MTT cytotoxicity assays were conducted to validate the anticancer effect of PSMA/Me/Fe_3_O_4_/PTX on MCF-7 and Skov-3 cells. The data indicated a significant difference between the control group and treatment groups, either PTX or PSMA/Me/Fe_3_O_4_/PTX treated MCF-7 cells after 24 and 48 h treatment (*p* < 0.05). For instance, as compared to the control group, PTX decreased cell viability in treatment groups (*p* < 0.05). In addition, there was a significant difference between 1 µM group and 4 and 8 µM groups (*p* < 0.05) (Fig. 4A). In MCF-7 cells treated with PSMA/Me/Fe_3_O_4_/PTX, there was a significant a decrease in cell viability value of all groups compared to control group (*p* < 0.05). The cell viability of 4 and 8 µM groups was low compared to 1 and 2 µM (*p* < 0.05). After 48 h treatment, compared to the control group, the cell viability was decreased in all groups treated with PTX and PSMA/Me/Fe_3_O_4_/PTX (*p* < 0.05) (Fig. 4A). Moreover, the cell viability of the 8 µM group was low compared to 1, 2, and 4 µM groups (*p* < 0.05). The same results were obtained in Skov-3 cells (Fig. 4A). For example, PTX reduced cell viability significantly in 2, 4, and 8 µM cells compared to control cells (*p* < 0.05) after 24 h (Fig. 4A). Furthermore, there was a significant difference between 1 µM group and groups treated with 2 and 4 µM PTX (*p* < 0.05). The cell viability of the 8 µM group was low when compared to 1, 2, and 4 µM groups (*p* < 0.05). Meanwhile, PSMA/Me/Fe_3_O_4_/PTX decreased cell viability in all groups after 24 h (*p* < 0.05). Similar to MCF-7 cells, the cell viability of 4 and 8 µM groups was low compared to 1 and 2 µM in Skov-3 cells (*p* < 0.05). There was a significant difference between the 4 µM group and the group treated with 8 µM PSMA/Me/Fe_3_O_4_/PTX (*p* < 0.05). As shown in Fig. 4A, both PTX and PSMA/Me/Fe_3_O_4_/PTX significantly led to a reduction in cell viability of Skov-3 cells after 48 h treatment (*p* < 0.05). There was a non-significant difference between the control group and the 1 µM group in the PTX treatment panel (*p* < 0.05). In cells treated with PSMA/Me/Fe_3_O_4_/PTX, compared to the 1 µM group, there was a significant decrease in cell viability of either the 4 or 8 µM group (*p* < 0.05). The IC_50_ values of PSMA/Me/Fe_3_O_4_/PTX for MCF-7 cells were 0.8 and 0.44 μM for 24 and 48 h, respectively. For Skov-3 cells, the values were 1.5 and 1.06 μM for 24 and 48 h, respectively. Table 1 displays the IC_50_ values for PTX and PSMA/Me/Fe_3_O_4_/PTX in cells. The curves of IC_50_ concentration of PTX and PSMA/Me/Fe_3_O_4_/PTX for 24 and 48 h were presented in Fig. 4B.

**Table 1:** IC_50_ values of PTX and PSMA/Me/Fe_3_O_4_/PTX for MCF-7 and Skov-3 cells

	MCF-7 cells		Skov-3 cells	
	24 h	48 h	24 h	48 h
PTX	0.99 µM	0.95 µM	1.9 µM	1.5 µM
PSMA/Me/Fe_3_O_4_/PTX	0.8 µM	0.44 µM	1.32 µM	1.06 µM

## Discussion

**Figure 5:**
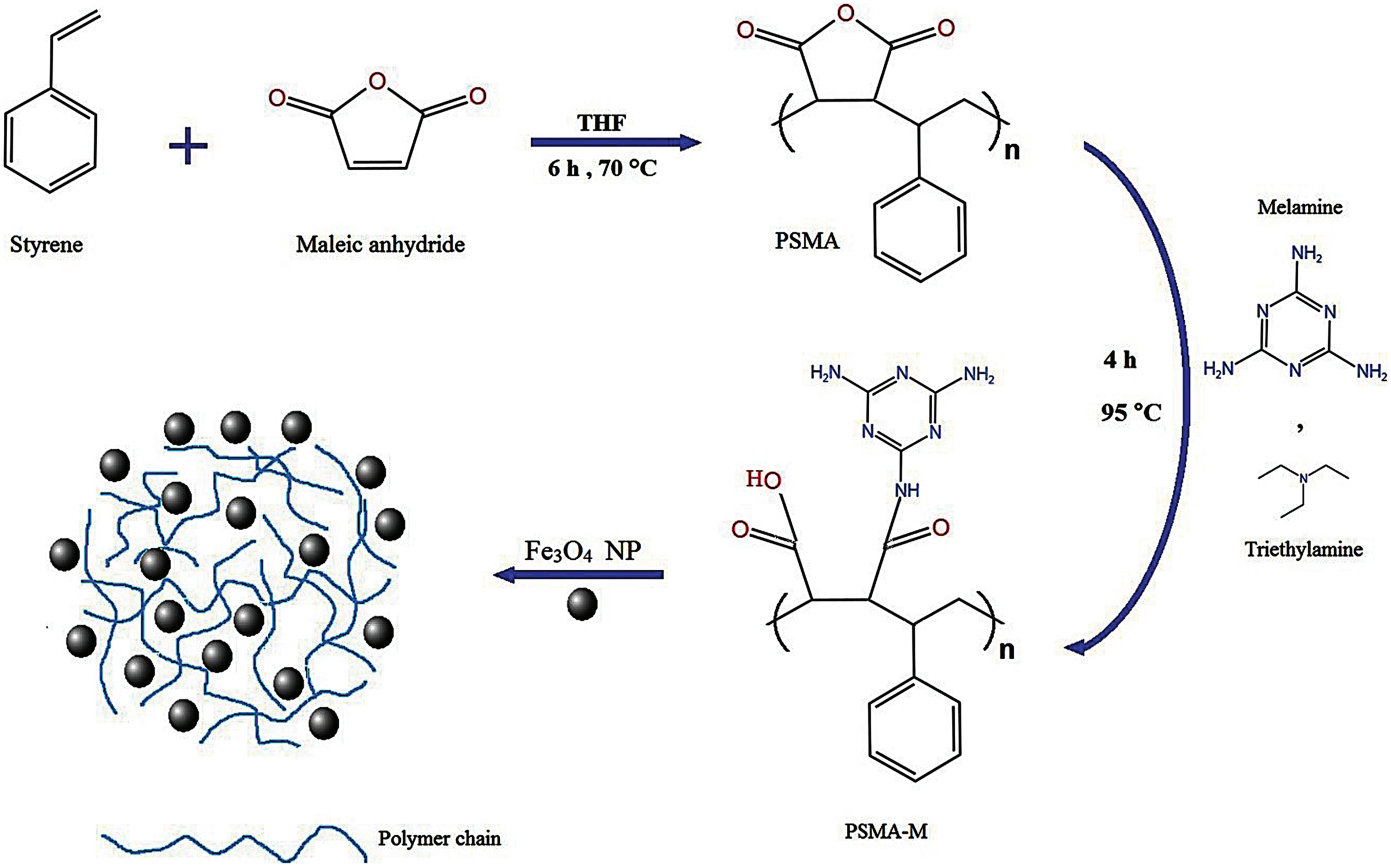
The general synthesis procedure of PSMA/Me/Fe_3_O_4_/PTX.

This study showed the synthesis of the PSMA/Me in two steps. First, ST and MA as initial monomers were copolymerized using free radical copolymerization in the presence of BPO as initiator. Next, a specific amount of Me as a graft agent and cross-linker was added to obtain the final polymeric product. PSMA/Me were loaded with Fe_3_O_4_ to form PSMA/Me/Fe_3_O_4_. In the final step, we loaded PTX into PSMA/Me/Fe_3_O_4_ to construct PSMA/Me/Fe_3_O_4_/PTX (Fig. 5). The synthesized copolymer was characterized using FT-IR, SEM, TGA, and XRD analyses. We used Me for our material because, among thermosetting polymers, Me is one of the hardest and stiffest existing polymer systems, which provides outstanding scratch resistance and surface gloss as well as good performance and appearance [8]. Thus, Me is used to improve the mechanical properties, moisture resistance, or fire resistance in many applications [8].

In XRD analysis, shifts in peak positions could signify the interactions between the polymer matrix and the magnetite nanoparticles. Furthermore, the relative intensities of the peaks provide information about the phase abundance and distribution within the composite. Variations in peak intensities may indicate differences in crystallite sizes or preferential orientation of crystalline domains. According to the PSMA-Fe_3_O_4_ spectrum in IR analysis, the width of the peaks in the region of 3000 to 3500 cm^−1^ has broadened due to the O-H group, and the peak has appeared in the region of 580 cm^−1^ is related to the Fe-O group in Fe_3_O_4_. It confirms the establishment of Fe_3_O_4_ nanoparticles on PMSA. PTX encapsulation in nanoparticles based on polymer metals is attracting more attention because it increases internalization and consequently promotes cytotoxicity and apoptosis in cancer cells [26]. The use of this copolymer is interesting because PSMA is a low-cost commercially accessible copolymer containing reactive groups in the central structure for additional functionalization [27].

Then we investigated the cytotoxicity of PTX and PSMA/Me/Fe_3_O_4_/PTX on two various cancer cell lines including MCF-7 cells and Skov-3 cells for 24 and 48 h. Our finding showed that PTX and PSMA/Me/Fe_3_O_4_/PTX decreased cell viability in both cells after 24 and 48 h dose/time-dependently, indicating inhibitory effects on cell proliferation. When PTX was loaded into PSMA/Me/Fe_3_O_4_/PTX, cell viability values were significantly decreased. Previous studies have shown that nanocarriers can deliver drugs into cells effectively and cause profound cytotoxicity. Through this system, cancer cells can not generally pump out drugs and fail to resistance against drugs. Similar to these results, in another study, we found that PSMA/Me/Fe_3_O_4_/PTX had a profound cytotoxic effect on human breast cancer cells including, MCF-7 and MDA-MB-231 cells (data not published). In a similar vein, Molaparast et al. synthesized the PSMA copolymer targeted with folic acid and then encapsulated doxorubicin into them. They showed that this drug delivery system had significant cytotoxic effects when exposed to human colorectal cells compared to free doxorubicin [28]. Similar to our polymer, Hasanzadeh et al. synthesized PSMA networks as a nano-chelating resin for the uptake of heavy metal ions [22]. They used this polymer for the adsorption of several ions such as Fe (II), Cu (II), Zn (II), and Pb (II).

In our previous study, we used PSMA as drug delivery for controlled drug delivery of ceftriaxone antibiotics [23]. *In vitro*, release test indicated that the amount of drug release in chemical loading was higher than the physical loading. In addition, a similar polymer was prepared by Nazarzadeh Zare and co-workers to deliver anti-antioxidant and heavy metal sorbent activity applications [27]. They declared that this polymer had antioxidant activity and heavy metals removal, which can be used in biomedical and industrial applications. It was demonstrated that the PTX-loaded polymeric nanoparticles based on α-tocopheryl succinate were successfully uptake by head and neck squamous cell carcinoma leading to cellular cytotoxicity [29]. In addition, the antitumor activity results showed that compared to free PTX, PTX-loaded polymeric nanoparticles exhibited much higher antitumor efficacy and apoptosis-inducing [29].

In an *in vitro* and *in vivo* study, the polymer-coated magnetic nanoparticles (Fe_3_O_4_) were designed for targeted delivery of PTX for fibrosarcoma therapy [30]. The results showed that SPION@Cs-PTX-PEG-FA had a spherical shape, suitable physical stability, desirable size, and charge. This polymer suppressed growth and promoted apoptosis of tumor cells. The reports of the *in vivo* study indicated that SPION@Cs-PTX-PEG-FA significantly decreased tumor size compared to free PTX and control groups, causing longer survival, and significantly increased splenocyte proliferation and IFN-γ levels [30]. Akbarian et al. designed a green synthesis of a new ZnO nanocarrier with PTX as a drug delivery system with high cytotoxicity against breast cancer cell line (MCF-7) and low side effects on normal cell line (fibroblast). PTX-loaded ZnO-Ch nanoparticles showed cytotoxic effects on MCF-7 cells with minimal harmful effects on normal fibroblasts. Also, the results of the apoptosis assay were consistent with the findings of MTT. Overall, ZnO-Ch nanoparticles can be used as a promising drug delivery platform for PTX with low side effects on normal cell lines and high cytotoxic effects on breast cancer cell lines [31].

In keeping, IC_50_ values showed that PSMA/Me/Fe_3_O_4_/PTX were more cytotoxic than free PTX because they could kill cancer cells at low concentrations (Table 1). Therefore, it may be assumed that PSMA/Me/Fe_3_O_4_/PTX successfully penetrates the cell membrane and distributes PTX inside the cell. A previous study showed that PTX nanoformulation causes a profound reduction in IC_50_ value of PTX-loaded solid lipid nanoparticles compared to free PTX [32,33]. A growing body of evidence has shown when therapeutic agents are incorporated into nanoparticles they have effective anticancer properties. Because nanocarriers can overwhelm the drug-resistance mechanisms of cancer cells [31]. Following endocytosis, in the cytoplasm, drugs are released to the cytoplasm, which in turn harm cellular organelles. This drug delivery is effective because it carries the epitome concentration of drugs at the cancer site while lessening systematic toxicity [34].

Overall, these findings showed the anticancer potential of PSMA/Me/Fe_3_O_4_/PTX and their application as a novel drug delivery system. We think that the PSMA/Me/Fe_3_O_4_/PTX may have the potential clinical application because it is a low-cost commercially accessible copolymer and can deliver drugs to cancer cells. It could kill both types of cancer cells (breast and ovary), which makes it a suitable carrier for drugs. As shown in Fig. 4, as a result, the PTX release from this polymer in neutral media was higher than in other media, suggesting application in physiological conditions.

However, the generalizability of these results is subject to certain limitations. For example, the effects of different morphologies of Fe_3_O_4_ or composite materials on drug EE and LE should be measured in further studies. In addition, other cell lines such as normal cells should be considered in further studies. The size and zeta potential of this carrier should be measured in further studies. In addition, *in vivo* models are required to validate our results, therefore, for clinical translation of these results further *in vivo* studies are recommended. We recommend studying cellular signaling behind the cytotoxicity of these particles.

## Conclusion

PSMA/Me/Fe_3_O_4_ was successfully synthesized and PTX was loaded to form PSMA/Me/Fe_3_O_4_/PTX. This drug delivery system showed profound cytotoxic effects on ovarian and breast cancer cells over a period of 24 and 48 h when compared to free PTX. Therefore, PSMA/Me/Fe_3_O_4_/PTX can be advanced into a novel formulation to significantly enhance the anticancer drug PTX. This formulation of PTX may be helpful in future pre-clinical cancer therapy.

## Data Availability

The datasets generated during and/or analyzed during the current study are available from the corresponding author on reasonable request.

## References

[1] Elumalai K, Srinivasan S, Shanmugam A. Review of the efficacy of nanoparticle-based drug delivery systems for cancer treatment. Biomed Technol. 2024;5:109–22. doi:10.1016/j.bmt.2023.09.001.

[2] Garbayo E, Pascual-Gil S, Rodríguez-Nogales C, Saludas L, Estella-Hermoso de Mendoza A, Blanco-Prieto MJ. Nanomedicine and drug delivery systems in cancer and regenerative medicine. Wiley Interdiscip Rev: Nanomed Nanobiotechnol. 2020;12(5):e1637; 32351045 10.1002/wnan.1637

[3] Salmanpour M, Yousefi G, Mohammadi-Samani S, Abedanzadeh M, Tamaddon AM. Hydrolytic stabilization of irinotecan active metabolite (SN38) against physiologic pH through self-assembly of conjugated poly (2-oxazoline)-poly (l-amino acid) block copolymer: a-synthesis and physicochemical characterization. J Drug Deliv Sci Tech. 2020;60:101933. doi:10.1016/j.jddst.2020.101933.

[4] Beach MA, Nayanathara U, Gao Y, Zhang C, Xiong Y, Wang Y, et al. Polymeric nanoparticles for drug delivery. Chem Rev. 2024;124(9):5505–616. doi:10.1021/acs.chemrev.3c00705; 38626459 PMC11086401

[5] Baranello MP, Bauer L, Benoit DS. Poly (styrene-alt-maleic anhydride)-based diblock copolymer micelles exhibit versatile hydrophobic drug loading, drug-dependent release, and internalization by multidrug resistant ovarian cancer cells. Biomacromolecules. 2014;15(7):2629–41. doi:10.1021/bm500468d; 24955779

[6] Li X, McTaggart M, Malardier-Jugroot C. Synthesis and characterization of a pH responsive folic acid functionalized polymeric drug delivery system. Biophys Chem. 2016;214:17–26. doi:10.1016/j.bpc.2016.04.002; 27183249

[7] Wilson RC, Pfohl WF. Study of cross-linking reactions of melamine/formaldehyde resin with hydroxyl functional polyester by generalized 2-D infrared spectroscopy. Vib Spectrosc. 2000;23(1):13–22. doi:10.1016/S0924-2031(99)00072-7.

[8] Merline DJ, Vukusic S, Abdala AA. Melamine formaldehyde: curing studies and reaction mechanism. Polym J. 2013;45(4):413–9. doi:10.1038/pj.2012.162.

[9] Gawde KA, Sau S, Tatiparti K, Kashaw SK, Mehrmohammadi M, Azmi AS, et al. Paclitaxel and di-fluorinated curcumin loaded in albumin nanoparticles for targeted synergistic combination therapy of ovarian and cervical cancers. Coll Surf B: Biointerfaces. 2018;167:8–19. doi:10.1016/j.colsurfb.2018.03.046; 29625422

[10] Tang H, Chen J, Wang L, Li Q, Yang Y, Lv Z, et al. Co-delivery of epirubicin and paclitaxel using an estrone-targeted PEGylated liposomal nanoparticle for breast cancer. Int J Pharm. 2020;573:118806. doi:10.1016/j.ijpharm.2019.118806; 31678519

[11] Lu Q, Gao W, Chen Z, Liu Z, Wang J, Zeng L, et al. Co-delivery of Paclitaxel/Atovaquone/Quercetin to regulate energy metabolism to reverse multidrug resistance in ovarian cancer by PLGA-PEG nanoparticles. Int J Pharm. 2024;655:124028. doi:10.1016/j.ijpharm.2024.124028; 38518871

[12] Wu J. The enhanced permeability and retention (EPR) effect: the significance of the concept and methods to enhance its application. J Pers Med. 2021;11(8):771. doi:10.3390/jpm11080771; 34442415 PMC8402171

[13] Korbecki J, Bosiacki M, Barczak K, Łagocka R, Brodowska A, Chlubek D, et al. Involvement in tumorigenesis and clinical significance of CXCL1 in reproductive cancers: breast cancer, cervical cancer, endometrial cancer, ovarian cancer and prostate cancer. Int J Mol Sci. 2023;24(8):7262. doi:10.3390/ijms24087262; 37108425 PMC10139049

[14] Momenimovahed Z, Tiznobaik A, Taheri S, Salehiniya H. Ovarian cancer in the world: epidemiology and risk factors. Int J Women’s Health. 2019;11:287–99. doi:10.2147/IJWH. 31118829 PMC6500433

[15] Bray F, Laversanne M, Sung H, Ferlay J, Siegel RL, Soerjomataram I, et al. Global cancer statistics 2022: GLOBOCAN estimates of incidence and mortality worldwide for 36 cancers in 185 countries. CA: a Cancer J Clin. 2024;74(3):229–63; 38572751 10.3322/caac.21834

[16] Salve R, Kumar P, Chaudhari BP, Gajbhiye V. Aptamer tethered bio-responsive mesoporous silica nanoparticles for efficient targeted delivery of paclitaxel to treat ovarian cancer cells. J Pharm Sci. 2023;112(5):1450–9. doi:10.1016/j.xphs.2023.01.011; 36669561

[17] Ribaux P, Wuillemin C, Petignat P, Delie F, Cohen M. NANO-SBT-PEDF delivery system: a promising approach against ovarian cancer? Heliyon. 2023;9(2):e13676. doi:10.1016/j.heliyon.2023.e13676; 36873150 PMC9975102

[18] Cheng X, Xie Q, Sun Y. Advances in nanomaterial-based targeted drug delivery systems. Front Bioeng Biotechnol. 2023;11:1177151. doi:10.3389/fbioe.2023.1177151; 37122851 PMC10133513

[19] Siegel RL, Miller KD, Jemal A. Cancer statistics, 2018. CA: a Cancer J Clin. 2018;68(1):7–30; 29313949 10.3322/caac.21442

[20] Rezaei S, Babaei M. A systematic literature review on direct and indirect costs of triple-negative breast cancer. Cost Eff Resour Alloc. 2023;21(1):92. doi:10.1186/s12962-023-00503-2; 38037138 PMC10688084

[21] Xu AP, Xu LB, Smith ER, Fleishman JS, Chen Z-S, Xu X-X. Cell death in cancer chemotherapy using taxanes. Front Pharmacol. 2024;14:1338633. doi:10.3389/fphar.2023.1338633; 38249350 PMC10796453

[22] Hasanzadeh R, Najafi Moghadam P, Samadi N. Synthesis and application of modified poly (styrene-alt-maleic anhydride) networks as a nano chelating resin for uptake of heavy metal ions. Polym Adv Technol. 2013;24(1):34–41. doi:10.1002/pat.v24.1.

[23] Moghadam PN, Azaryan E, Zeynizade B. Investigation of poly (styrene-alt-maleic anhydride) copolymer for controlled drug delivery of ceftriaxone antibiotic. J Macromol Sci, Part A: Pure Appl Chem. 2010;47(8):839–48. doi:10.1080/10601325.2010.492265.

[24] Gurenko VE, Tolstoy VP, Gulina LB. The effect of microtube formation with walls, containing Fe_3_O_4_ nanoparticles, via gas-solution interface technique by hydrolysis of the FeCl_2_ and FeCl_3_ mixed solution with gaseous ammonia. Nanosyst: Phys, Chem, Math. 2017;8(4):471–5.

[25] Abbasi R, Nejati V, Rezaie J. Exosomes biogenesis was increased in metformin-treated human ovary cancer cells; possibly to mediate resistance. Cancer Cell Int. 2024;24(1):137. doi:10.1186/s12935-024-03312-6; 38627767 PMC11022479

[26] Mahdi SA, Kadhim AA, Albukhaty S, Nikzad S, Haider AJ, Ibraheem S, et al. Gene expression and apoptosis response in hepatocellular carcinoma cells induced by biocompatible polymer/magnetic nanoparticles containing 5-fluorouracil. Electron J Biotechnol. 2021;52:21–9. doi:10.1016/j.ejbt.2021.04.001.

[27] Nazarzadeh Zare E, Mansour Lakouraj M, Najafi Moghadam P, Hasanzadeh R. Novel conducting nanocomposite based on polypyrrole and modified poly (styrene-alt-maleic anhydride) via emulsion polymerization: synthesis, characterization, antioxidant, and heavy metal sorbent activity. Polym Compos. 2015;36(1):138–44. doi:10.1002/pc.v36.1.

[28] Molaparast M, Ehsanimehr S, Kahyaei M, Mahboubi N, Shafiei-Irannejad V. Polymeric complex based on poly (styrene-alt-maleic anhydride)-targeted with folic acid for doxorubicin delivery to HT-29 colorectal cancer cells. Int J Polym Mat Polym Biomat. 2023;72(3):181–93. doi:10.1080/00914037.2021.1999953.

[29] Riestra-Ayora J, Sánchez-Rodríguez C, Palao-Suay R, Yanes-Díaz J, Martín-Hita A, Aguilar MR, et al. Paclitaxel-loaded polymeric nanoparticles based on α-tocopheryl succinate for the treatment of head and neck squamous cell carcinoma: *in vivo* murine model. Drug Deliv. 2021;28(1):1376–88. doi:10.1080/10717544.2021.1923863; 34180747 PMC8245075

[30] Al-Obaidy R, Haider AJ, Al-Musawi S, Arsad N. Targeted delivery of paclitaxel drug using polymer-coated magnetic nanoparticles for fibrosarcoma therapy: *in vitro* and *in vivo* studies. Sci Rep. 2023;13(1):3180. doi:10.1038/s41598-023-30221-x; 36823237 PMC9950487

[31] Akbarian M, Mahjoub S, Elahi SM, Zabihi E, Tashakkorian H. Green synthesis, formulation and biological evaluation of a novel ZnO nanocarrier loaded with paclitaxel as drug delivery system on MCF-7 cell line. Coll Surf B: Biointerfaces. 2020;186:110686. doi:10.1016/j.colsurfb.2019.110686; 31816463

[32] Pi C, Zhao W, Zeng M, Yuan J, Shen H, Li K, et al. Anti-lung cancer effect of paclitaxel solid lipid nanoparticles delivery system with curcumin as co-loading partner *in vitro* and *in vivo*. Drug Deliv. 2022;29(1):1878–91. doi:10.1080/10717544.2022.2086938; 35748365 PMC9246235

[33] Mohammad IS, He W, Yin L. A smart paclitaxel-disulfiram nanococrystals for efficient MDR reversal and enhanced apoptosis. Pharm Res. 2018;35:1–18. doi:10.1007/s11095-018-2370-0; 29488114

[34] Das CGA, Kumar VG, Dhas TS, Karthick V, Kumar CMV. Nanomaterials in anticancer applications and their mechanism of action—a review. Nanomed: Nanotechnol, Biol Med. 2023;47:102613. doi:10.1016/j.nano.2022.102613; 36252911

